# The Association Between Living Area in Childhood and Respiratory Disease Mortality in Adulthood

**DOI:** 10.3389/ijph.2022.1604778

**Published:** 2022-10-07

**Authors:** Ayumu Iwasaki, Masayuki Teramoto, Isao Muraki, Kokoro Shirai, Akiko Tamakoshi, Hiroyasu Iso

**Affiliations:** ^1^ Public Health, Department of Social Medicine, Osaka University Graduate School of Medicine, Suita, Japan; ^2^ Public Health, Department of Preventive Medicine, Hokkaido University Graduate School of Medicine, Sapporo, Japan

**Keywords:** mortality, cohort study, all-cause mortality, childhood living area, large city, industrial area, respiratory disease, cox proportional hazard model

## Abstract

**Objective:** No studies have examined the association between characteristics of urban areas and future respiratory disease mortality. We examined whether the type of living area during childhood was associated with all-cause and respiratory disease mortality in adulthood.

**Methods:** A total of 81,413 Japanese participants aged 40–79 years old completed a lifestyle questionnaire including the type of childhood living areas. The Cox proportional hazards regression model was used to calculate the multivariable hazard ratios (HRs) with 95% confidence intervals (CIs) of all-cause and respiratory disease mortality.

**Results:** Living in large city areas in childhood was associated with a higher risk of all-cause mortality [HR = 1.05 (95% CI, 1.01–1.10)], but not with respiratory disease mortality [HR = 1.04 (95% CI, 0.92–1.18)] compared to rural and remote areas. The excess risk of all-cause and respiratory disease mortality was primarily found in industrial areas among men; the respective multivariable HRs were 1.28 (95% CI, 1.00–1.64) and 1.90 (95% CI: 1.10–3.29).

**Conclusion:** Eliminating childhood health hazards associated with living in industrial areas suggested to reduce the risk of mortality from respiratory diseases in adulthood.

## Introduction

According to the 2018 Revision of World Urbanization Prospects, approximately 55% of the global population live in urban areas. This proportion is expected to increase to 68% by 2050 [1].

In the global burden of disease (GBD) study from 2017, the prevalence of respiratory diseases is 7.1%, with chronic obstructive pulmonary disease (COPD) at 3.9% and asthma at 3.6%. About 3.9 million annual deaths and 1,470 disability-adjusted life years (DALYs) per 100,000 are attributable to respiratory diseases worldwide [2]. Smoking is the most common cause of COPD among men. Household air pollution from solid fuels in South Asia and Sub-Saharan Africa; exposure to household and outdoor air pollutants in Southeast Asia, Oceania, East Asia, North Africa, and the Middle East; and smoking in other regions are the most common causes of COPD for women. Since urban industrial areas are at a relatively higher risk of air pollution and lower socioeconomic status (SES), living in these areas during childhood can increase the risk of respiratory diseases [3, 4].

Air pollution, such as nitrogen dioxide, PM 2.5, or PM 10 is a major risk factor for respiratory disease in industrialized areas [5, 6]. In three different cohorts of 2,120 children in Southern California, air concentrations of nitrogen dioxide (NO2), PM2.5, PM10, and Ozone were measured at outdoor monitoring stations in the target communities [7]. After four years of follow-up, reductions in pollution concentrations of NO2, PM2.5, and PM10 were associated with improved respiratory function, regardless of pre-existing asthma. Due to the impaired respiratory function and increased risk of childhood respiratory disease, higher exposure to air pollution in childhood can also result in an increased risk of incident respiratory diseases in adulthood [7].

Furthermore, childhood SES—including educational level, passive smoking, and malnutrition in the early stages of life—has been associated with childhood respiratory diseases such as asthma and wheezing, and respiratory disease mortality in adulthood [8–10]. Since urbanization leads to increased exposure to air pollutants [3, 11], living in an urban environment during childhood is expected to result in increased mortality from respiratory diseases in adulthood. However, little is known about the impact of living in urban areas during childhood on respiratory health outcomes in adulthood. To the best of our knowledge, no studies have examined the association between characteristics of urban areas and future respiratory disease mortality.

Since Japan has a history of serious air pollution from the 1900s with remarkable modernization, there is a great need for scientific evidence to research the effect of childhood living environment on lifelong health [12]. Therefore, we aimed to examine whether the type of living area in childhood was associated with all-cause and respiratory disease mortality in adulthood in a large long-term cohort study of Japanese men and women.

## Methods

### Study Population

The Japan Collaborative Cohort Study for Evaluation of Cancer Risk (JACC) study was conducted between 1988 and 1990. The study screened 110,585 participants, including 46,395 men and 64,190 women aged 40–79 years old, from 45 communities across Japan. The methodology of the JACC study has been described elsewhere [13].

In brief, the participants responded to self-administered questionnaires regarding their sociodemographic characteristics, medical history, and lifestyle. From 110,585 participants, we excluded 29,172 (12,048 men and 17,124 women) in eight areas because the question on the living area during childhood was not included in their questionnaires. Thus, 81,413 participants (34,347 men and 47,066 women) were included in the analyses.

Before completing the questionnaires, the participants or community representatives were asked for informed consent to participate in this epidemiological study. Informed consent was obtained from each participant in 29 of the 37 study areas (written consent in 28 areas and oral consent in one area). In the remaining eight areas, group consent was obtained from each area leader. The ethics committees approved the investigation protocol of Nagoya University (The number of the ethics approval: 227), Osaka University (14285-8), and Hokkaido University (I14-044).

### Assessment of the Type of Living Areas During Childhood

We asked participants, “Which area did you live in the longest during the 12 years before you graduated from elementary school?”. In Japan, elementary school graduation is at age 12. In the present study, therefore, childhood was defined as 0–12 years of age. The answers included “large cities with a population of 500,000 or more,” “other cities with a population less than 500,000 (middle city),” “rural and fishing villages,” “remote islands and remote areas,” and “others”. When choosing “large cities with a population of 500,000 or more,” the participants answered one of the following five options: residential, commercial, industrial, mixed city, or other city areas. The mixed city area was defined as the areas with mixed characteristics of residential, commercial, and industrial areas. Areas with characteristics that did not fit into any of these categories were classified as other city areas. Rural and fishing villages, remote islands, and remote areas were combined as rural and remote areas because of the small sample size per area. Finally, we classified their responses into the following seven groups: rural and remote, middle city, residential, commercial, industrial, mixed city, and other city areas.

### Assessment of Covariates

We obtained the other information about sociodemographic and lifestyle factors from a self-administered questionnaire at baseline. These included age, sex, adulthood variables of educational level, past medical history such as hypertension and diabetes, height, weight, smoking and alcohol drinking status, exercise and walking habits, mental status, employment status, and eating habits, and childhood passive smoking status. Body mass index was estimated as the body weight (kg) divided by the height squared (m^2^).

### Mortality Surveillance

Mortality surveillance in each community was systematically conducted by reviewing the death certificates in each area. Mortality data were reported centrally to the Ministry of Health and Welfare through the local public health center. The underlying causes of death were coded based on the 10th revision of the International Classification of Diseases (ICD-10). Follow-up was continued in 37 areas until the end of 1999 in four areas, 2003 in three areas, 2008 in two areas, and 2009 in the rest of the areas. In this study, the endpoint of death was all-cause (ICD-10 codes: all) and respiratory diseases (ICD-10 codes: J01-J99). The date of moving from the community was verified through population registration documents. When participants moved out, they were treated as censored cases.

### Statistical Analysis

Person-years of follow-up were calculated from the date of study enrollment to the date of death, emigration from the community, or the end of follow-up, whichever happened first. The analysis of covariance was used to test for differences in age-adjusted means and the prevalence of baseline characteristics. Hazard ratios (HRs) with 95% confidence intervals (CIs) of all-cause and respiratory disease mortality were calculated using Cox proportional hazards regression models according to the childhood living area category. We confirmed no violation for the proportional hazard assumption in all models. We adjusted for age (continuous), sex (women or men), educational level (≤18 or ≥19 years of age upon completion of education), history of hypertension (yes or no) and diabetes (yes or no), body mass index (sex-specific quintile), smoking status (never, ex-smoker, current smoker of 1–19, or current smoker of ≥20 cigarettes per day), childhood passive smoking status (yes, no, or uncertain), alcohol consumption (never drinker, ex-drinker, current drinker of 0.1–45.9, or ≥46.0 g ethanol per day), hours of exercise (almost never, 1–4 h, or ≥5 h per week) and walking (almost never, 0.5 h, or >0.5 h per day), perceived mental stress (low, moderate, or high), employment status (unemployed or employed), frequency of consuming vegetables, fish, fruits, and soybean intakes (quintile). SAS version 9.4 (SAS, Inc., Cary, NC) was used for all statistical analyses.

## Results

The age-adjusted baseline characteristics of the participants are presented according to the type of childhood living areas in Table 1. The overall mean age was 57.7 years, and the overall percentage of men, history of hypertension, diabetes, and smoking status were 42.2%, 22.9%, 5.4%, and 25.8%, respectively. Compared to rural and remote areas, participants living in industrial areas in childhood were more likely to smoke, feel mental stress, and be unemployed. They were also less likely to eat vegetables and fruits, exercise, and receive higher education.

**TABLE 1:** Age-adjusted baseline characteristics of participants according to childhood living areas [The Japan Collaborative Cohort Study, 1988, Japan].

	Type of living areas during childhood							Overall
	Rural and remote areas	Middle city areas	Large city areas					
			Residential areas	Commercial areas	Industrial areas	Mixed city areas^a^	Other city areas^b^	
No. of participants	62,037	5,397	9,228	1,811	397	1,659	884	81,413
Age, y	58.0	55.4	56.9	58.7	59.2	57.3	57.5	57.7
Sex,% of men	44.0	38.8	34.2	37.2	47.4	37.4	39.4	42.2
Body mass index, kg/m^2^	22.8	22.8	22.7	22.7	23.0	22.7	22.8	22.8
History of hypertension,%	22.7	24.2	23.4	22.7	21.9	23.5	21.0	22.9
History of diabetes mellitus,%	5.1	5.5	6.6	7.7	5.9	7.3	5.4	5.4
Current smoker,%	26.1	23.5	24.9	27.1	33.0	25.7	27.8	25.8
Current drinker,%	39.9	38.9	39.1	42.5	39.4	41.1	39.6	39.8
History of childhood passive smoking,%	72.2	73.6	74.7	78.3	75.4	75.9	74.3	72.8
High mental stress,%	20.8	23.5	23.1	27.3	27.2	25.1	17.4	21.5
College or higher education,%	11.8	21.6	16.4	22.7	7.0	14.5	10.1	13.3
Unemployed,%	19.3	20.6	22.2	18.6	25.4	21.8	26.2	19.9
Walking≥30 min/day,%	52.7	42.8	42.8	41.2	45.4	40.9	48.2	50.1
Exercise≥1 h/week,%	6.0	5.8	4.6	5.1	4.0	4.0	5.4	5.7
Vegetable intake, times/week	2.1	2.1	2.0	2.0	1.9	1.9	2.0	2.1
Fish intake, times/week	2.1	1.8	1.8	1.8	1.8	1.7	2.0	2.0
Fruits intake, times/week	2.0	2.2	2.3	2.3	1.9	2.2	2.0	2.0
Soybeans intake, times/week	1.9	2.0	1.8	2.0	1.8	1.8	2.0	1.9

Data are mean for continuous variables and percentages for categorical variables.

BMI indicates body mass index.

a Mixed city areas: areas with characteristics of residential, commercial, and industrial area.

b Other city areas: areas with characteristics that did not fit into any of residential, commercial, and industrial areas.

### Risk of All-Cause Mortality According to the Type of Childhood Living Areas

During the median follow-up period of 18.8 years, we documented a total of 18,700 deaths (10,769 men and 7,931 women) from all causes and 2,367 deaths (1,584 men and 783 women) from respiratory disease. Table 2 shows the HRs of all-cause mortality according to the type of childhood living areas. As shown in Table 2, living in large city areas during childhood was associated with a higher risk of all-cause mortality than those in rural and remote areas; the multivariable HR was 1.05; (95% CI, 1.01–1.10). Similar positive associations were observed for men and women, but was only statistically significant for men; the multivariable HRs were 1.08 (1.02–1.15) for men and 1.03 (0.96–1.09) for women. In large city areas, the excess risk was primarily found in industrial areas: the HR of living in industrial areas during childhood was 1.26 (1.05–1.52). Again, similar positive associations were found between men and women, but was only statistically significant among men; the multivariable HRs were 1.28 (1.00–1.64) for men and 1.22 (0.92–1.63) for women. Living in mixed city areas during childhood was associated with an increased risk of all-cause mortality only for men; the multivariable HRs were 1.19 (1.03–1.38) for men and 0.99 (0.84–1.18) for women. Such associations were not observed in middle city, residential, or commercial areas.

**TABLE 2:** Hazard ratios (95% confidence intervals) of all-cause mortality according to childhood living areas [The Japan Collaborative Cohort Study, 1988, Japan].

Type of living areas	Person-years	Number of deaths	Mortality rate (1,000 person-years)	Age- and sex- adjusted hazard ratio (95% confidence interval)		Multivariable hazard ratio (95% confidence interval)^a^	
Total							
Rural and remote areas	994,524	15,027	15.1	1.00		1.00	
Middle city areas	85,315	951	11.1	1.00 (0.93–1.06)		1.02 (0.96–1.09)	
Large city areas							
Residential areas	137,904	1,664	12.1	1.04 (0.99–1.09)	1.07 (1.02–1.11)^b^	1.03 (0.98–1.08)	1.05 (1.01–1.10)^b^
Commercial areas	26,818	404	15.1	1.04 (0.94–1.15)		1.04 (0.94–1.14)	
Industrial areas	5,816	111	19.1	1.30 (1.08–1.57)		1.26 (1.05–1.52)	
Mixed city areas	23,976	317	13.2	1.10 (0.99–1.23)		1.09 (0.98–1.22)	
Other city areas	14,082	226	16.0	1.17 (1.03–1.33)		1.12 (0.98–1.28)	
Men							
Rural and remote areas	426,471	8,820	20.7	1.00		1.00	
Middle city areas	32,336	505	15.6	0.99 (0.90–1.08)		1.01 (0.92–1.11)	
Large city areas							
Residential areas	46,887	845	18.0	1.06 (0.99–1.14)		1.04 (0.97–1.12)	
Commercial areas	9,567	228	23.8	1.13 (0.99–1.28)		1.09 (0.96–1.25)	
Industrial areas	2,422	64	26.4	1.30 (1.02–1.66)	1.11 (1.05–1.17)^b^	1.28 (1.00–1.64)	1.08 (1.02–1.15)^b^
Mixed city areas	8,501	181	21.3	1.22 (1.05–1.41)		1.19 (1.03–1.38)	
Other city areas	5,265	126	23.9	1.21 (1.02–1.45)		1.15 (0.96–1.37)	
Women							
Rural and remote areas	568,053	6,207	10.9	1.00		1.00	
Middle city areas	52,979	446	8.4	1.01 (0.92–1.11)		1.03 (0.94–1.14)	
Large city areas							
Residential areas	91,017	819	9.0	1.02 (0.95–1.10)	1.02 (0.96–1.08)^b^	1.02 (0.95–1.10)	1.03 (0.96–1.09)^b^
Commercial areas	17,252	176	10.2	0.95 (0.82–1.10)		0.99 (0.85–1.15)	
Industrial areas	3,395	47	13.8	1.31 (0.99–1.75)		1.22 (0.92–1.63)	
Mixed city areas	15,474	136	8.8	0.98 (0.82–1.16)		0.99 (0.84–1.18)	
Other city areas	8,817	100	11.3	1.12 (0.92–1.36)		1.12 (0.92–1.37)	

a Adjusted for age, sex, educational level, history of hypertension, history of diabetes, body mass index, smoking status, childhood passive smoking status, alcohol consumption, hours of exercise, hours of walking, perceived mental stress, regular employment, and dietary intakes of vegetable, fish, fruits and soybeans.

b Hazard ratios for large city areas.

### Risk of Respiratory Disease Mortality According to the Type of Childhood Living Areas

Table 3 shows the HRs of respiratory disease mortality according to the type of childhood living area. Living in large city areas during childhood was not associated with a higher risk of respiratory disease mortality than in rural or remote areas. In large city areas, the excess risk was primarily observed in industrial areas for men, but not for women; the multivariable HRs were 1.90 (1.10–3.29) for men and 0.87 (0.28–2.70) for women. Living in other city areas during childhood was associated with an increased risk of respiratory disease mortality; the HR was 1.42 (1.01–1.99). Such associations were not observed in middle, residential, commercial, or mixed city areas.

**TABLE 3:** Hazard ratios (95% confidence intervals) of respiratory disease mortality according to childhood living areas [The Japan Collaborative Cohort Study, 1988, Japan].

Type of living areas	Person-years	Number of deaths	Mortality rate (1,000 person-years)	Age- and sex- adjusted hazard ratio (95% confidence interval)		Multivariable hazard ratio (95% confidence interval)^a^	
Total							
Rural and remote areas	994,524	1944	2.0	1.00		1.00	
Middle city areas	85,315	100	1.2	0.90 (0.74–1.10)		0.93 (0.76–1.14)	
Large city areas							
Residential areas	137,904	190	1.4	1.03 (0.89–1.20)	1.07 (0.95–1.21)^b^	0.99 (0.85–1.16)	1.04 (0.92–1.18)^b^
Commercial areas	26,818	50	1.9	1.01 (0.77–1.34)		1.00 (0.75–1.32)	
Industrial areas	5,816	16	2.8	1.54 (0.94–2.52)		1.56 (0.95–2.56)	
Mixed city areas	23,976	32	1.3	0.96 (0.68–1.36)		0.95 (0.67–1.35)	
Other city areas	14,082	35	2.5	1.48 (1.06–2.06)		1.42 (1.01–1.99)	
Men							
Rural and remote areas	426,471	1,312	3.1	1.00		1.00	
Middle city areas	32,336	67	2.1	0.96 (0.75–1.23)		0.99 (0.77–1.27)	
Large city areas							
Residential areas	46,887	119	2.5	1.09 (0.91–1.32)	1.13 (0.97–1.31)^b^	1.02 (0.84–1.23)	1.07 (0.92–1.24)^b^
Commercial areas	9,567	33	3.4	1.07 (0.76–1.51)		1.02 (0.72–1.45)	
Industrial areas	2,422	13	5.4	1.84 (1.07–3.18)		1.90 (1.10–3.29)	
Mixed city areas	8,501	19	2.2	0.94 (0.60–1.47)		0.91 (0.58–1.43)	
Other city areas	5,265	21	4.0	1.41 (0.92–2.17)		1.39 (0.90–2.14)	
Women							
Rural and remote areas	568,053	632	1.1	1.00		1.00	
Middle city areas	52,979	33	0.6	0.79 (0.56–1.12)		0.84 (0.59–1.20)	
Large city areas							
Residential areas	91,017	71	0.8	0.94 (0.73–1.20)	0.99 (0.81–1.21)^b^	0.94 (0.73–1.21)	1.01 (0.82–1.23)^b^
Commercial areas	17,252	17	1.0	0.93 (0.57–1.50)		1.01 (0.62–1.63)	
Industrial areas	3,395	3	0.9	0.93 (0.30–2.88)		0.87 (0.28–2.70)	
Mixed city areas	15,474	13	0.8	1.00 (0.58–1.73)		1.03 (0.59–1.79)	
Other city areas	8,817	14	1.6	1.58 (0.93–2.69)		1.53 (0.89–2.61)	

a Adjusted for age, sex, educational level, history of hypertension, history of diabetes, body mass index, smoking status, childhood passive smoking status, alcohol consumption, hours of exercise, hours of walking, perceived mental stress, regular employment, and dietary intakes of vegetable, fish, fruits and soybeans.

b Hazard ratios for large city areas

## Discussion

In this large Japanese cohort study, we found that living in large city areas during childhood was associated with a higher risk of all-cause mortality, but not of respiratory disease mortality, compared to rural and remote areas. On the other hand, the excess risks of all-cause and respiratory disease mortality in adulthood were primarily observed in men who are living in industrial areas among large city areas during childhood. These associations remained statistically significant after adjusting for potential confounders in adulthood. To the best of our knowledge, this is the first study to find a positive association between living in industrial city areas during childhood and mortality from respiratory disease.

Our results were supported by the finding from a previous study on the effect of a child’s environment on the development of respiratory function; the growth deficits of the forced expiratory volume in one second (FEV1) between 10 and 18 years old were 105.8 ml by acid vapor (*p* = 0.004), 101.4 ml by NO_2_ (*p* = 0.005), 79.7 ml by PM2.5 (*p* = 0.04), and 87.9 ml by elemental carbon (*p* = 0.007) in eleven air polluted areas, compared to the least polluted area of Los Angeles [6].

Childhood SES was associated with educational attainment and adulthood SES, and subsequently, lung disease during adulthood [8, 14, 15]. In a cohort study of 28,132 men and women aged ≥20 years old based on two longitudinal population studies in Denmark, the association between SES and respiratory disease-related adult mortality was examined [16]. In that study, the components of SES included income, number of people per room in the house, use of central heating as an indicator of house quality and dampness, educational level, employment status, and type of residence. During a mean follow-up of 12 years, only education level was significantly associated with the risk of respiratory disease mortality. People with a higher education level (≥8 years) had a lower risk of respiratory disease mortality compared to those with a lower education level (<8 years); the odds ratios adjusting for potential adulthood covariates were 0.63 (95% CI, 0.47–0.86) among women and 0.72 (0.55–0.95) among men. These results suggest that low childhood SES, primarily low education level, can increase the risk of mortality from respiratory disease in adulthood. In the present study, the proportion of people who received higher education was lowest in the industrial areas (7.0%) compared to other areas (10.1%–22.7%).

Air pollution, passive smoking, and malnutrition in childhood can cause impaired lung function or chest illness in childhood, resulting in adult respiratory disease [17]. In the cohort of 197,263 Omani men and women aged 1–20 years old, the association between living near industrial areas and respiratory disease was examined [18]. High, intermediate, and low (as reference) exposure villages were defined according to the distance from major refineries in the industrial area: ≤5, >5 to 10, and ≥20 km. The relative risks of asthma and acute respiratory diseases were 2.3 (95% CI, 2.1–2.6) and 3.7 (95% CI, 3.1–4.5) for intermediate exposure zones; 2.6 (95% CI, 2.3–2.8) and 3.6 (95% CI, 3.0–4.4) for high exposure zones. In a cohort of 10,069 Japanese children aged 6–9 years without a history of asthma, exposure to elemental carbon, but not nitrogen oxide, was associated with a higher risk of incident asthma; the odds ratios were 1.07 (1.01–1.14) and 1.01 (0.99–1.03), respectively [19]. Japan has a history of severe air pollution with significant modernization since the 1900’s. The air pollution was declined during the World War Ⅱ, but appeared again with the industrial recovery after the War [12, 20]. Children growing up in large cities, more specifically industrial areas during that period, were more likely to be exposed to air pollution, resulting in impaired lung development and deaths from respiratory diseases in adulthood [21, 22].

Passive smoking during childhood influences lung function and development and risk for future respiratory diseases in children. Abnormal growth and inflammation of small airways from passive smoking during the postnatal period may account for increased cough, poor lung function, airway resistance, and a higher fraction of exhaled nitric oxide level [23–25]. A cohort study of 70,900 non-smoking men and women in the United States examined the association between passive smoking in childhood and mortality from all-causes, ischemic heart disease, stroke, and COPD in adulthood. Living with one smoker for 16–18 years during childhood was significantly associated with a 34% increased risk of mortality from COPD in adulthood compared to living with non-smokers, but not with total mortality, stroke, or ischemic heart disease [9].

The strengths of the present study are its prospective study design, sufficient sample size, and long follow-up duration to investigate the association after adjustment for many potential confounders.

However, this study had several limitations. First, we obtained information on childhood living areas through self-reports, potentially resulting in false reports. Second, we did not have detailed information, such as the duration of living in industrial areas during childhood, level of air pollution, and childhood SES. Further research is warranted to elucidate the detailed mechanisms of living in large cities and industrial areas during childhood on the risk of respiratory diseases. Finally, since the present results reflect conditions in Japan prior to the 1960’s, caution should be exercised when applying the present results to current situation in urban industrial areas.

In conclusion, living in large cities and industrial areas during childhood was positively associated with all-cause mortality. The excess risk of respiratory disease mortality was also observed among men living in industrial areas in childhood after adjusting for potential confounding factors in adulthood. The present study suggests that eliminating childhood health hazards associated with living in industrial areas may reduce the risk of respiratory diseases in adulthood. Further studies are needed to create healthy environments for children to prevent lifelong disease.

## Abbreviations

JACC, Japan Collaborative Cohort; CI, confidence interval; GBD, global burden of disease; COPD, chronic obstructive pulmonary disease; SES, socioeconomic status; HR, hazard ratio; ICD-10, The 10th revision of the International Classification of Diseases; DALYs, disability-adjusted life years; FEV1, the forced expiratory volume in 1 s; NO_2_, Nitrogen dioxide.
